# Arthroscopic treatment of non-homogeneous calcifying tendinitis of the rotator cuff

**DOI:** 10.1186/s40064-016-1792-6

**Published:** 2016-02-27

**Authors:** George El Rassi, Jihad Matta, Georges Haidamous, Patrik Brogard, Philipe Clavert, Jean-Francois Kempf, Jihad Irani

**Affiliations:** Department of Orthopaedic Surgery, Saint George Hospital, P.O. Box 166378, Ashrafieh, Beirut, 1100 2807 Lebanon; Department of Orthopaedic Surgery, Illkirch Hospital, Strasbourg, France; Faculty of Medicine, University of Balamand, Beirut, Lebanon

**Keywords:** Calcifying tendinitis, Arthroscopic, Calcification excision, Acromioplasty

## Abstract

The purpose of this study is to investigate the clinical outcome of arthroscopic treatment for patients with non-homogeneous infiltrated calcifying tendinitis of the rotator cuff (type III), and to assess the optimal method for this arthroscopic treatment. We retrospectively reviewed the charts of 81 patients who underwent arthroscopic treatment for non-homogeneous infiltrated calcifying tendinitis of the rotator cuff (type III). Patients were divided into two groups: Group A (n = 31) consisted of patients who underwent excision of calcification, and Group B comprised patients who underwent acromioplasty alone (n = 50). The clinical outcome of treatment was assessed using Constant-Murley score. Twenty-three of the 81 patients were males and 58 were females. The mean duration of symptoms from onset to the first clinic visit was 3.88 years (SD ± 3.06 years). The right side was involved in 47 patients, the left side in 34 patients, and none had bilateral involvement. Patients from Group B had higher 16 improvement of their Constant-Murley score (from 48.96 to 88.06) when 17 compared to group A (from 45.39 to 67.23). Treatment of type III calcifying tendinitis is different than type I and II. Subacromial decompression may be considered in all patients suffering from type III non-homogeneous infiltrated calcifying tendinitis of the shoulder.

## Background

Calcifying tendinitis (CT) is a common disorder of the rotator cuff affecting 2.7 to 20 % of asymptomatic subjects (Bosworth 1941; Uhthoff et al. 2006; Seil et al. 2006) and about 17 % of painful shoulders (DePalma 1961). Women are generally more affected than men and the right or the dominant shoulder is more affected than the left.

The etiology of CT is somehow controversial. Some authors described it as a degenerative process of the tendon fibers with secondary dystrophic calcifications. Uhthoff et al. (2006) provided morphological evidence of a formative and a resorptive phase of calcific deposits. During the formative phase, and through a multifocal, active, cell-mediated process, the tendinous tissue is transformed into fibrocartilage, which is later, calcified. This phase is usually painless. Once formed, the calcific deposits enter a resting stage and they maybe symptomatic. After a variable period of time, macrophages and multinuclear giant cells start an inflammatory reaction and the deposits enter the resorptive phase. This stage may be very painful.

**Fig. 1:**
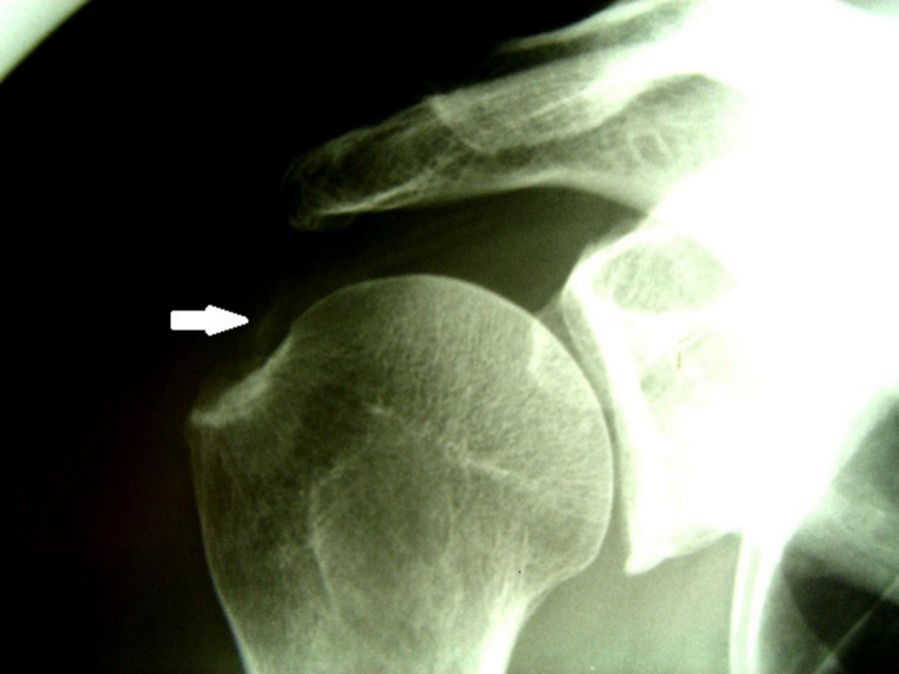
AP X-ray of the shoulder showing a type III calcifying lesion. *White arrow* pointing at the soft contour of the linear calcifications infiltrating the tendon

Based on their radiological appearance, Gartner el. Al. classified calcifying tendinitis into three types (Gartner and Simons 1990). In type 1, the deposits are well demarcated and dense, in type II the deposits are either dense with soft contours or transparent with sharp contours and type III, the deposits are transparent with soft contours (Fig. 1). Type III deposits are usually infiltrated and they resemble the resorptive phase of the disease.

Treatment for symptomatic patients with calcifying tendinitis is conservative (Cho et al. 2010) especially during the formative sub-acute stages of the disease (Re and Karzel 1993). Injections with corticosteroids combined with local anesthetic agents are often effective adjuvant. Shockwave therapy can be used as part of the conservative therapy or as part of an neo-adjuvant treatment prior to surgical excision leading to improved outcomes compared to surgery alone (Jiménez-Martín et al. 2012). When conservative therapy fails in terms of symptom control and the deposits don’t show any sign of spontaneous resolution, open or arthroscopic surgery is warranted. Most authors agree that complete excision of the calcium deposits with or without acromioplasty and or bursectomy should be the goal of arthroscopic treatment (Porcellini et al. 2004).

The aim of this study is to detail the arthroscopic treatment for patients with type III calcifying tendinitis, and to report their clinical outcomes, in order to clarify the optimal surgical approach for this subcategory of patients.

## Methods

This study was based on reviewing the medical charts and radiographs of all patients at our orthopedic department who underwent shoulder arthroscopy for calcifying tendinitis of the rotator cuff between January 1999 and January 2010. All surgeries were performed by the same surgeon (the PI). The cases of calcifying tendinitis were classified according to Gartner et al. into types I, II, or, III based on their radiological appearance (Gartner and Simons 1990). The inclusion criteria consisted of patients with chronic shoulder pain and evidence of Gartner’s type III calcific tendinitis on shoulder radiographic examinations; also, patients who had at least 2 years of follow-up.

All patients with type II or I calcifications, previous open or arthroscopic surgery of the shoulder and patients with osteoarthritis, rotator cuff tear, or acromioclavicular joint pathology were excluded from the study.

Approval from the institution ethical committee was obtained. During preoperative evaluation, patients were questioned about the period of non-operative treatment, their demographics, and any medical or surgical history. True antero-posterior views in neutral, internal, and external rotation and trans-scapular roentgenography were obtained for each patient.

### Pre and postoperative

Radiographs were evaluated by the same radiologist for the type and localization of rotator cuff calcific tendinitis and for any other associated lesions. Patients were followed up every 6 weeks for at least 2 years to assure regression of symptoms and disappearance of calcification on radiographs following arthroscopy. Radiographs were taken 2 years post op. The Constant-Murley score was used before and 2 years after surgery to assess clinical outcome and estimate shoulder function (Constant and Murley 1987). From a total of 411 shoulders (384 patients) treated by shoulder arthroscopy for several categories of calcification, 81 patients met our inclusion criteria for the study. 23 patients were males (28.4 %). The average age at diagnosis was 55 years (SD ± 9.2 years). Most of the patients were referred for surgery following a prolonged period of non-operative treatment with mean duration of 3.88 years (SD ± 3.0 years). The conservative therapy consisted mainly of non-steroidal anti-inflammatory medications, physical therapy, and steroid injections. The right side was involved in 47 (58 %) patients and the left side in 34 (42 %) patients. None of the patients had bilateral shoulder involvement. The dominant side was affected in 72 (88.9 %) patients. The initial characteristics of patients are summarized in Table 1.

**Table 1:** Demographic data of the patients

Baseline characteristics	Resection of calcification (n = 31) Group A	Acromioplasty (n = 50) Group B	P value
Age (years) (mean + SD)	55.1 + 7.5	55.5 + 10.1	0.857
Age (years) (median)	57	56.5	0.105
Age (years) (range)	42–69	33–81	0.6470.079
Male (%)	12 (38.7)	11 (22.0)	0.965
Side, right (%)	17 (54.8)	30 (60.0)	0.959
Dominant, R (%)	25 (80.6)	47 (94)	0.857
Follow-up, months (mean + SD)	71 + 1.9	78 + 2.1	0.073
Duration of pain (years) (mean + SD)	4.2 + 3.4	3.7 + 2.7	0.105

### Surgical technique

**Fig. 2:**
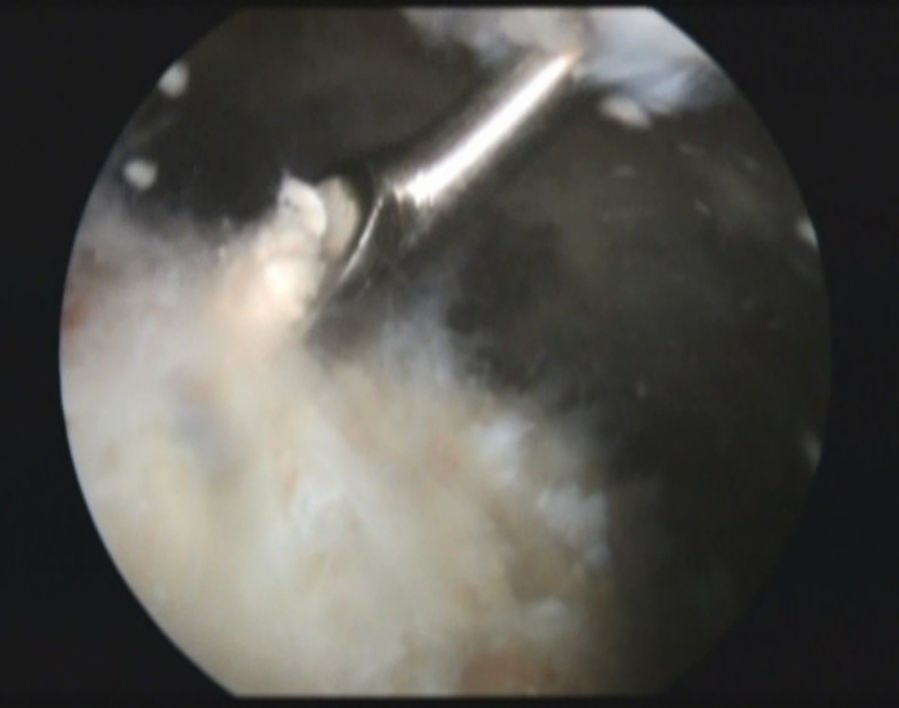
Posterior portal arthroscopic view showing a curette inserted through a lateral portal scrapping off a white chalky calcific lesion from the substance of the supraspinatus tendon

All patients were placed in beach-chair position for arthroscopy with routine instrumentation and draping. The gleno-humeral joint was explored through a standard posterior portal, using a 4.5-mm, 30-degree arthroscope. An anterolateral portal was then made for irrigation. After evaluation of the gleno-humeral joint, the subacromial space was entered via the posterior portal in a standard manner. A lateral trans-deltoid instrumental approach was then created, after which a bursectomy was performed to allow for better exploration of the rotator cuff. A spinal needle introduced via the lateral approach was used to localize the calcium deposits. Exploration of the calcium deposits was accomplished using a lateral to medial approach, followed by an anterior to posterior method, by rotating the arm during the arthroscopic procedure. Identification of the calcium deposits was confirmed by the appearance of small granular “snowflakes” at the tip of the needle. After recognizing these calcification deposits, a small scalpel blade was used to incise the rotator cuff in the direction of the fibers, and a small curette was used to gently scrape away the calcium deposits (Fig. 2). Once the calcific deposits were evacuated, irrigation was performed to avoid leaving any calcium deposits in the subacromial space. To avoid rotator cuff tearing, patients with calcifications that are too infiltrated within the tendon tissue were left alone; acromioplasty with acromioclavicular ligament release was done instead.

### Statistical analysis

Numerical and categorical data were expressed as the mean standard deviation (SD) or median with range and percentages, respectively. Continuous variables in demographic data, baseline characteristics, and the results were analyzed by Student’s t test or Mann–Whitney test when appropriate. Chi square and Fisher’s exact test were used for comparing categorical data. All the statistical analysis was completed using the IBM SPSS statistics 20 program (IBM corporation ©), with a significant P value specified as <0.05.

## Results

Sixty-nine shoulders (85.2 %) had calcifications in one tendon (the supra-spinatus or the infra-spinatus), whereas 12 (14.8 %) had calcifications in two tendons (the infra- and supra-spinatus) Table 2. For the purpose of statistical analysis, and in an attempt to determine the therapeutic value of acromioplasty and excision of calcification, shoulders that underwent surgery were divided into two groups: Group A (n = 31) consisted of shoulders that underwent bursectomy and excision of calcification, and group B (n = 50) comprised shoulders in which bursectomy, coraco-acromial ligament release and acromioplasty was performed. There were no statistical differences between the two groups in terms of age, gender, and duration of symptoms before presentation. When comparing Constant-Murley scores postoperatively in each group, there was a statistically significant improvement of this score in all groups with a P value less than 0.001. Patients’ baseline Constant-Murley scores were almost similar pre-operatively. However, patients from Group B had the highest improvement of their Constant-Murley score post-operatively (from 48.96 to 88.06) compared to Group A (from 45.39 to 67.23). In other words, patients from group B had improved by 79.9 % in comparison with group A, who improved by only 48.1 % after surgery. These data are summarized in Table 3.

**Table 2:** Preoperative location of calcific tendinitis on perspective tendons

Tendon involved	Resection of calcification (n = 31) Group A	Acromioplasty (n = 50) Group B	P value
Supraspinatus (%)	26 (83.9)	40 (80.0)	0.376
Infraspinatus (%)	2 (6.5)	1 (2.0)	0.959
Supra + infraspinatus (%)	3 (9.7)	9 (18.0)	0.373

**Table 3:** Outcome and Constant-Murley score results

Outcomes	Resection of calcification (n = 31) Group A	Acromioplasty (n = 50) Group B	P value
Radiographic disappearance of calcifications, N (%)	24 (77.4)	37 (74)	0.729
Constant-Murley score			
Preoperative	45.39 13.5	48.96 16.6	0.203
Postoperative	67.23 10.2	88.06 6.4	<0.001

## Discussion

The treatment of patients with calcifying tendinitis is initially conservative and depends on the evolution of the disease. Most cases are typically managed with anti- inflammatory medications, physiotherapy, and local steroid injections; however open or arthroscopic surgery can be resorted to recalcitrant cases (Re and Karzel 1993; Kachewar and KulKarni 2013; Seyahi and Demirhan 2009). Our patients were referred to surgery after long period of non-operative treatment including non-steroidal anti-inflammatory medications and local injection of steroids.

**Fig. 3:**
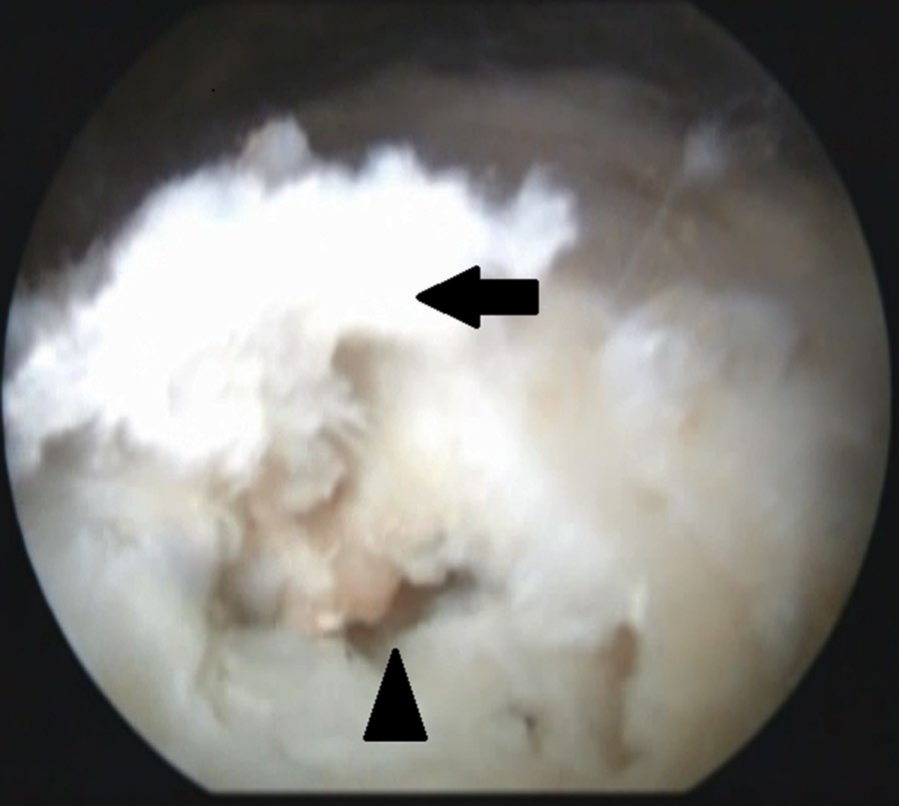
Lateral portal arthroscopic view showing a large defect in the supraspinatus tendon as a result of calcification scrapping. *Black arrow* pointing at part of the tendon that became detached due to scrapping and curettage of the lesion. *Black arrow* head pointing at the defect

The main goal of arthroscopic treatment for patients with CT is to remove the calcium deposits (Jerosch et al. 1998); nonetheless, removing the deposits in patients with type III infiltrated CT will require an aggressive debridement that would increase the risk of partial or even full-thickness tendon tears (Maier et al. 2013) (Fig. 3). When faced with deeply infiltrating deposits in our case series, the orthopedic surgeon chose to preserve the tendon’s integrity and leave the deposits alone.

The role of subacromial decompression is still controversial. A study by Mole et al. (1992) reported a cohort of 112 patients with calcifying tendinitis of the rotator cuff treated arthroscopically. They classified their patients into three types according to their radiological appearance, and then treated the patients by excision of the calcium deposit or acromioplasty, or both. They had a satisfactory clinical outcome in 82 % of their patients, yet they found that acromioplasty did not have a positive effect on the final clinical outcome. Nevertheless, they believed that acromioplasty might be indicated in only type C calcification, without giving any explanation of the reasons behind this belief.

Patte and Goutallier classified rotator cuff calcific tendinitis into two types: dense and homogeneous, and non-homogeneous infiltrated calcifying tendinitis (Patte and Goutallier 1987). They performed open or arthroscopic subacromial decompression on all patients, and no cases underwent any excision of calcium deposits. They found that patients who had heterogeneous infiltrated calcifying tendinitis had more satisfactory results after acromioplasty relative to patients with dense homogeneous calcific deposits.

Unlike type I or II calcification (wherein the calcium deposit is localized and superficial), type III calcification is more infiltrated and deeper in the tendon. The cause of pain in patients with type III CT is not clear. Uhthoff et al. (2006) proposed that it originates from increased intra-tendinous pressure in the vicinity of the calcium deposits. The cause of pain is a consequence of the inflammatory cascade that started with the beginning of the resorptive phase. Our subjective experience in this field led us to hypothesize that the tendon is unhealthy and diseased and consequently can’t function properly. This dysfunction of the rotator cuff may result in secondary impingement; therefore, acromioplasty is more effective than the removal of the calcific deposit alone in this subset of patients. Our understanding is that these patients suffer from shoulder pain mainly due to impingement; therefore, an associated acromioplasty may be indicated to ensure a more satisfactory outcome.

The disappearance of calcification seems to be higher in patients who underwent removal of their calcification (77 %) rather than isolated acromioplasty (74 %). However, this difference in calcification disappearance was not statistically significant (P = 0.729).

## Limitations

Our study has its limitations; the most obvious is its retrospective nature. We thought of reviewing our experience in managing infiltrated calcifying tendinitis of the rotator cuff, which is not very well described in the medical literature. Although the study is retrospective, it reflects our practice that has been standard over the study period. A prospective double-blinded study should be considered in the future. The use of only constant score in our pre and postop clinical evaluations is another limitation. Additional objective tests should have been employed. Also, the seldom dependence on radiography to evaluate calcium deposits pre and postoperatively is another limitation, especially when evaluating patients with type III calcifications.

## Conclusion

The arthroscopic treatment for type III non-homogeneous infiltrated calcifying tendinitis of the rotator cuff is different than that for type II and I. Arthroscopic sub-acromial decompression maybe considered as part of the treatment plan in this category of patients.
